# Supplementary material for: Semaphorin 3A-Neuropilin-1 Signaling Modulates MMP13 Expression in Human Osteoarthritic Chondrocytes
Source: Int J Mol Sci. 2022 Nov 16;23(22):14180. doi: 10.3390/ijms232214180 (PMC9699590; doi:10.3390/ijms232214180)
Supplement: Supplementary file 1 [file ijms-23-14180-s001.zip › ijms-2026273-supplementary.pdf]

Suppl. Files

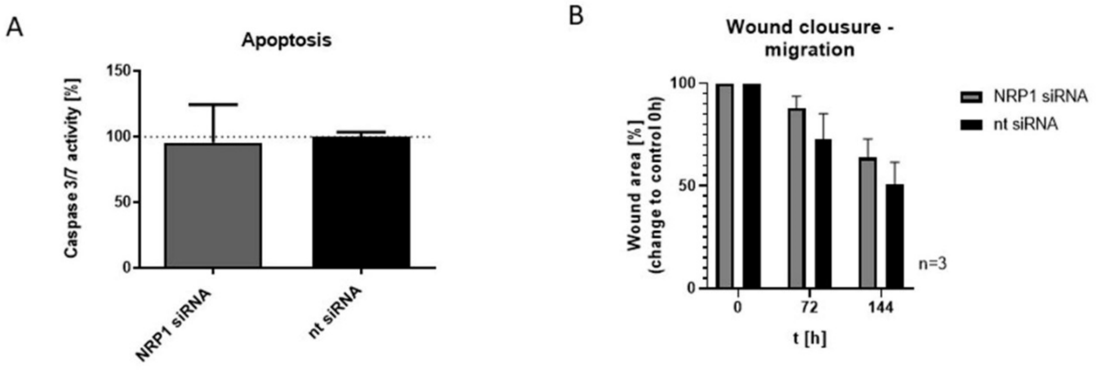

Figure S1: Effect of siRNA mediated knockdown on apoptosis and migration

A) Apoptosis was determined after transfection with *NRP1* siRNA or nt siRNA. One sample t-test, n= 5; B) Migration was determined after transfection with *NRP1* siRNA or nt siRNA. One sample t-test, n=4;

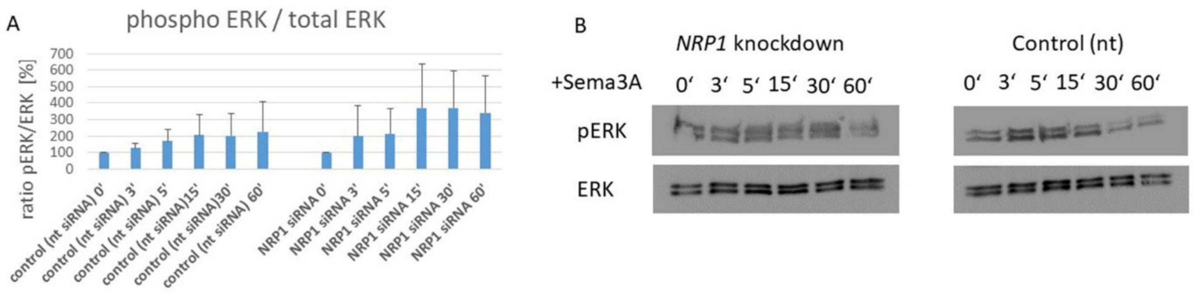

Figure S2: Effect of Sema3A on ERK phosphorylation in OA chondrocytes

A) Relative protein expression of pERK and ERK was determined densitometrically. The relative ratio of pERK / ERK is shown, n=3; B) Representative Western Blot image of phospho-ERK (pERK) and total-ERK (ERK) protein after stimulation of *NRP1* knockdown and control cells (nt) with 100ng/ml Sema3A for 0, 3, 5, 15, 30 and 60 minutes.
